# Controllable Charge Storability in InP/ZnSe Core/Shell Quantum Dots toward Bioinspired Optical Synaptic Application

**DOI:** 10.1002/advs.202511714

**Published:** 2025-11-05

**Authors:** Guohao Wen, Bingbing Huo, Dingting Zheng, Xiang Zheng, Guanlin Ke, Zhiguo Chi, Honglei Wu, Botao Ji, Zhenhua Sun

**Affiliations:** ^1^ Key Laboratory of Optoelectronic Devices and Systems of the Ministry of Education and Guangdong Province State Key Laboratory of Radio Frequency Heterogeneous Integration College of Physics and Optoelectronic Engineering Shenzhen University Shenzhen 518060 China; ^2^ Key Laboratory of 3D Micro/Nano Fabrication and Characterization of Zhejiang Province School of Engineering Institute of Advanced Technology Westlake Institute for Advanced Study Westlake University Hangzhou 310024 China

**Keywords:** charge storage, core–shell quantum dots, neuromorphic application, optoelectronic synapse, phototransistor

## Abstract

Optoelectronic materials with proper charge storage play a pivotal role in the development of artificial neuromorphic devices aiming to mimic the visual, sensory, and memory functions of the human nervous system. This study presents the controllable charge storability in Indium Phosphide quantum dots through being capped with Zinc Selenide shells of different thicknesses. The organic transistors with the quantum dots integrated demonstrate shell‐thickness‐dependent optoelectronic memory characteristics, featuring optically programmable‐electrically erasable channel states. Analysis reveals that the optoelectronic performance of the device is ascribed to the photoexcitation and the following charge storage process in the quantum dots. The device of the thickest quantum‐dot‐shell performs well as an optoelectronic synapse to emulate the entire human visual sensory and memory function. The frequency‐dependent synaptic potentiation/depression, paired‐pulse facilitation, short/long‐term memory, and “learning‐experience” behavior are exhibited in the optoelectronic synaptic device through optical stimuli manipulation. Moreover, the optical sensory performance of the device can be enhanced by a positive gate bias. It enables a successful emulation of Pavlov's dog classical conditioning experiments, realizing the associative learning characteristic with optical and electric signals. This work provides an effective solution for a stable and controllable charge storage medium for optoelectronic synapse applications.

## Graphical abstract

A field‐effect transistor incorporating type‐I InP/ZnSe core–shell quantum dots demonstrates light‐ and gate‐tunable synaptic plasticity. By leveraging field‐assisted photoinduced charge trapping, the device emulates essential neuromorphic functions, including paired‐pulse facilitation, memory consolidation, and classical conditioning. This work presents a promising platform for future neuromorphic visual systems and artificial cognitive hardware.

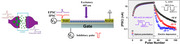

## 1 Introduction

Artificial neuromorphic devices, which aim to emulate the adaptive and energy‐efficient processing capabilities of biological neural systems, offer significant advantages in parallel data processing, in‐memory computing, and energy minimization—key requirements for applications such as autonomous perception, edge computing, and interactive robotics.^[^ ^1^, ^2^, ^3^ ^]^ Among the diverse hardware implementations explored to date, field‐effect transistor (FET)‐based artificial synapses represent a promising direction due to their compatibility with existing semiconductor technologies, ease of signal modulation, and versatility in material selection.^[^ ^4^, ^5^, ^6^ ^]^ A central mechanism underpinning synaptic behavior in such devices is the charge‐trapping effect, wherein charge carriers are captured and retained in dielectric or interfacial states, modulating the conductance of the channel. This mechanism enables essential features of biological synapses, such as short‐term and long‐term plasticity, bidirectional weight modulation, and non‐volatile memory storage.^[^ ^7^, ^8^, ^9^, ^10^, ^11^ ^]^ Moreover, when properly engineered, charge‐trapping‐based transistors can respond to multiple stimuli—electrical or optical—enabling multimodal synaptic functionalities that are critical for perception‐oriented neuromorphic systems, such as visual sensory units.^[^ ^11^, ^12^, ^13^ ^]^

In this context, colloidal quantum dots (CQDs) have emerged as a versatile class of nanomaterials that can uniquely serve as both the photoactive medium and the charge‐trapping reservoir. Due to their quantum‐confined energy structures, tunable surface states, and high defect tolerance, CQDs offer tailorable charge dynamics and spectral response ranges spanning the visible to infrared regimes.^[^ ^14^, ^15^ ^]^ Recent work has demonstrated that CQDs can be integrated into phototransistor architectures to realize optoelectronic synapses with optical plasticity and prolonged charge retention characteristics.^[^ ^8^, ^16^, ^17^ ^]^ These features make CQDs exceptionally suitable for neuromorphic visual sensing, where a device is expected not only to detect incident light but also to “remember” and “learn from” optical stimuli—a function that mimics the synaptic processes in retinal circuits. In particular, core–shell structured semiconductor quantum dots with a type‐I energy band alignment—where both electrons and holes are confined within the core—exhibit enhanced charge storage capability due to their ability to spatially isolate photogenerated carriers from environmental quenching, thereby prolonging retention times and enabling robust, non‐volatile synaptic behavior.^[^ ^8^, ^18^, ^19^, ^20^ ^]^

In this work, we demonstrate that the charge storage capability of Indium Phosphide–Zinc Selenide (InP/ZnSe) core–shell quantum dots (QDs) is significantly enhanced with increasing shell thickness. This behavior is investigated using a Poly(3‐hexylthiophene‐2,5‐diyl) (P3HT)‐based transistor integrated with QD films of varying shell configurations. The incorporation of QDs enables stable bidirectional programming of the device via both optical and electrical pulses, with excellent endurance exceeding 1000 cycles. Building on this functionality, the device is further utilized to emulate a range of optoelectronic synaptic behaviors, including frequency‐dependent potentiation/depression, paired‐pulse facilitation (PPF), short‐term and long‐term memory transition, as well as experience‐based learning behaviors. Additionally, it is found that the optical response of the device can be effectively enhanced by applying a positive gate bias. Leveraging this property, we successfully mimic Pavlov's classical conditioning, demonstrating associative learning behavior mediated by coupled optical and electrical inputs. Most reported artificial optoelectronic synapses employing quantum dots as charge‐trapping centers rely on a dielectric blocking layer to suppress charge relaxation.^[^ ^21^, ^22^, ^23^, ^24^, ^25^, ^26^ ^]^ The application of type‐I core–shell QDs can eliminate this requirement, simplifying device fabrication and potentially reducing operating energy and response time.^[^ ^27^, ^28^, ^29^ ^]^ Furthermore, the Pb‐free InP/ZnSe system used here offers environmental safety and long‐term stability. Overall, this work provides a validated and scalable strategy toward energy‐efficient and environmentally benign artificial optoelectronic synapses.

## 2 Results and Discussion

### 2.1 Charge Storage in QDs for Optoelectronic Memory Application

**Figure 1:**
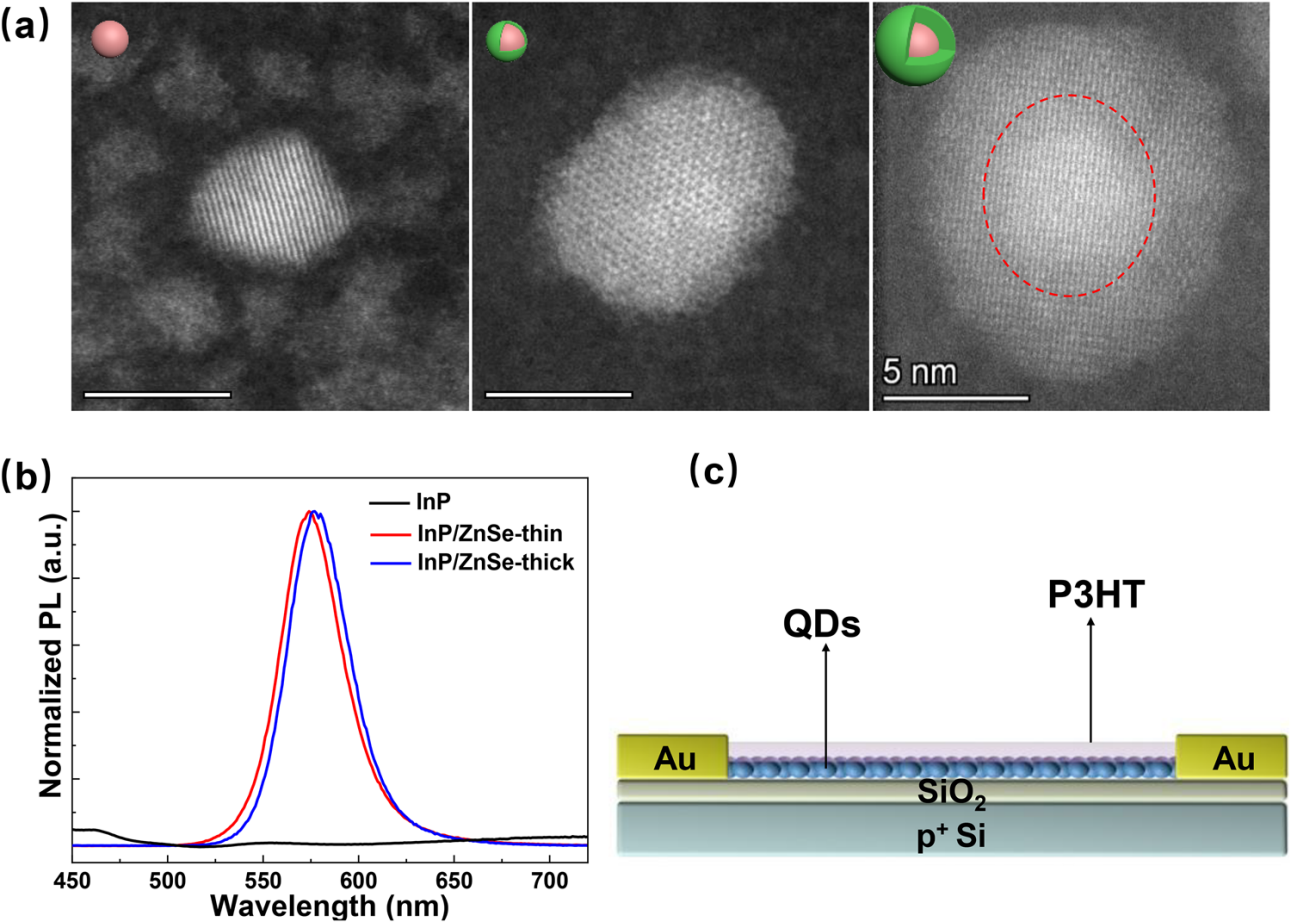
a) High‐angle annular dark‐field transmission electron microscopy (HAADF‐TEM) images of the InP, InP/ZnSe‐thin, and InP/ZnSe‐thick QDs from left to right. The schematic illustrations of structures are shown in the top‐left corners of the corresponding images. Three TEM images have the same scale bar of 5 nm. The red dashed line in the image of InP/ZnSe‐thick QD denotes the InP core; b) Photoluminescence (PL) spectra of the three types of QDs; c) Schematic illustrations of the P3HT‐based thin film transistor with QDs integrated into.

Three types of quantum dots (QDs) were synthesized using a well‐established method, as detailed in the Experimental Section. The synthesis began with the preparation of InP core QDs, followed by the growth of a ZnSe shell on their surfaces. By varying the shell growth duration, two core–shell structures with different shell thicknesses were obtained and are referred to as InP/ZnSe‐thin and InP/ZnSe‐thick QDs, respectively. High‐angle annular dark‐field transmission electron microscopy (HAADF‐TEM) images of the InP, InP/ZnSe‐thin, and InP/ZnSe‐thick QDs are presented in **Figure** **1**a, which are put on left, middle, and right, respectively. Their structures are schematically illustrated in the top‐left corners of each corresponding image. The pristine InP QD exhibits an average diameter of ≈4 nm. The size difference between the InP QD and the InP/ZnSe‐thin QD indicates a ZnSe shell thickness of ≈1.5 nm, while the InP/ZnSe‐thick QD has a shell thickness of ≈4 nm. Due to the very thin ZnSe shell in the InP/ZnSe‐thin QD, the shell cannot be clearly distinguished in the TEM image. As the shell thickness increases, the ZnSe lattice becomes more continuous and well‐defined. However, due to the ZnSe being grown at a high temperature of 340 °C, the QD shows a uniform shell layer without stacking faults in the crystalline phase.^[^ ^30^ ^]^ Therefore, the lattice difference between InP and ZnSe is not readily visible. Yet, the TEM image of the InP/ZnSe‐thick QD shows a clear core‐shell contrast because of the higher atomic number of the indium element, as denoted by the red dashed line.^[^ ^31^ ^]^ Moreover, the successful formation of the ZnSe shell is confirmed by both photoluminescence (PL) spectra (Figure 1b) and absorbance spectra (Figure S1a, Supporting Information). The bare InP QDs exhibit negligible PL emission, which is typically attributed to exciton quenching by surface trap states.^[^ ^32^ ^]^ Upon ZnSe shell growth, a prominent PL peak emerges around 574 nm, indicating effective surface passivation. As the shell becomes thicker, the PL peak exhibits a redshift, reflecting modifications to the exciton confinement environment. Similarly, in the absorbance spectra (Figure S1a, Supporting Information), the first excitonic peak of the InP QDs appears at 517 nm. With ZnSe shell capping, this peak also shifts to longer wavelengths, consistent with the exciton leakage effect—where the electron and hole wavefunctions partially delocalize into the shell. These combined spectral shifts further verify the successful and progressive capping of the ZnSe shell.^[^ ^33^, ^34^, ^35^ ^]^ Each type of QD was subsequently integrated into a P3HT‐based thin‐film transistor, as illustrated in Figure 1c. The devices were fabricated on Si/SiO_2_ substrates by sequential spin‐coating of the QD layer and the P3HT semiconductor. Two gold (Au) electrodes were thermally evaporated as the source and drain electrodes through a shadow mask to define a channel with a width/length of 2/0.2 mm. Further fabrication details are provided in the Experimental Section. For clarity, the transistors fabricated with different QDs are hereafter referred to as the InP‐device, InP/ZnSe‐thin‐device, and InP/ZnSe‐thick‐device, respectively.

**Figure 2:**
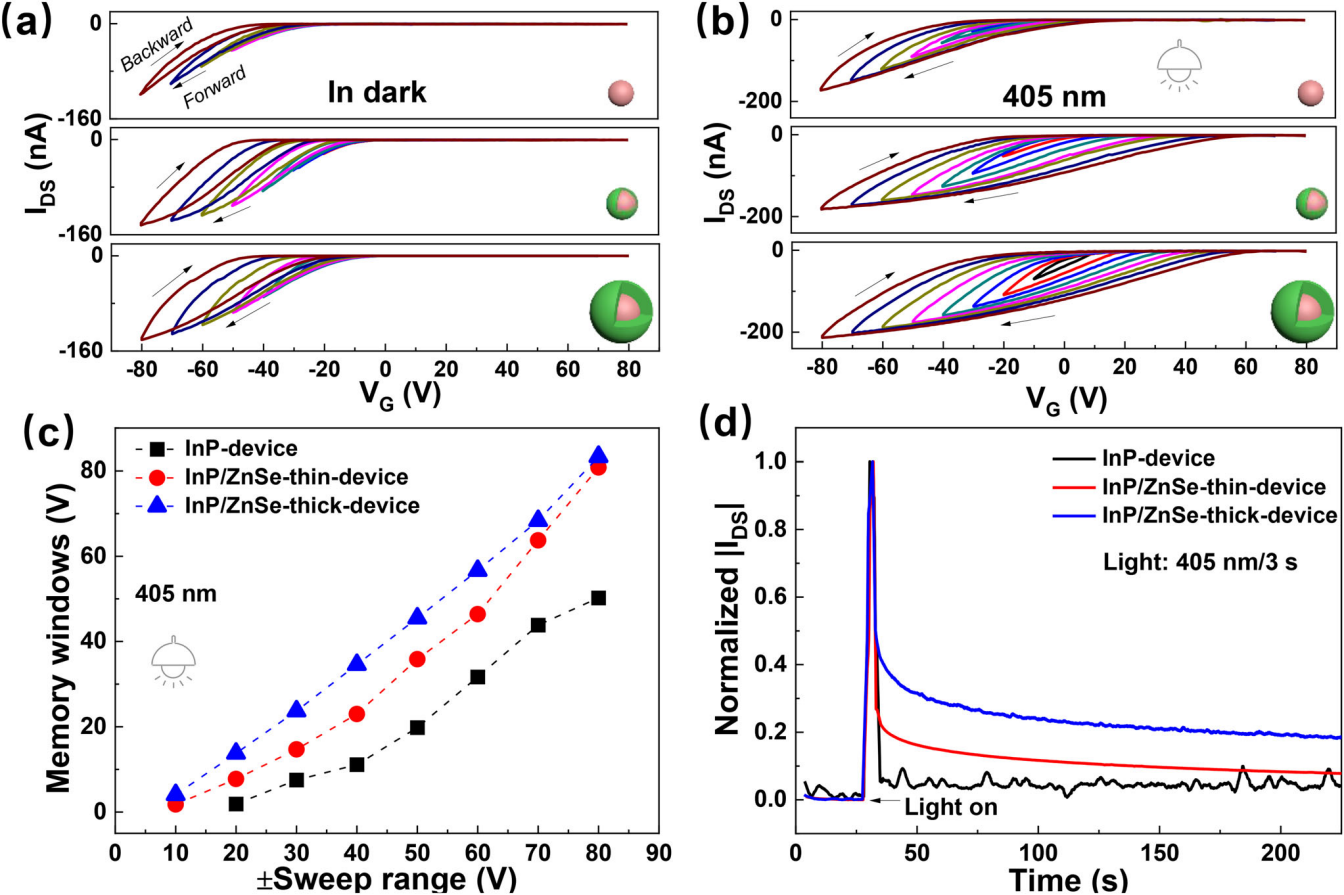
Cyclic transfer curves of the three QD‐integrated devices measured with varying V_G_ and V_DS_ = −30 V in the dark (a) and under 405 nm light illumination (b); c) Plots of memory windows as a function of the V_G_ sweep ranges extracted from (b); d) Dynamic response of I_DS_ after applying a light pulse. The I_DS_ is recorded at V_G_ = 0V and V_DS_ = −30 V.

The transfer characteristics of the devices were measured under various conditions with a V_DS_ of −30 V. **Figure** **2**a shows the cyclic transfer curves of the three QD‐integrated devices, measured in the dark with varying gate voltage (V_G_). In the measurement of each curve, the V_G_ was swept from positive to negative, defined as the forward sweep, and then back to positive, defined as the backward sweep. The curves were obtained sequentially, starting from the smallest sweep range to the largest. In all three devices, the transfer curves exhibit increasing hysteresis with wider sweep ranges, indicating the charge storage during the sweeping process. Furthermore, a clear trend is observed when comparing across devices: the hysteresis magnitude increases with the shell thickness, implying that thicker ZnSe shells facilitate more pronounced charge storage within the QDs.^[^ ^8^, ^36^, ^37^ ^]^ To confirm the role of QDs in this effect, a control device without QDs (P3HT‐device) was fabricated and characterized under the same measurement protocol, as shown in Figure S2 (Supporting Information). The cyclic transfer curves in Figure S2a (Supporting Information) reveal that the P3HT‐device exhibits negligible hysteresis, confirming that the observed charge storage behavior in the QD‐devices originates from the presence of the QDs themselves. For further comparison, the transfer measurements were repeated under 405 nm light illumination, and the results are presented in Figure 2b. To better quantify the hysteresis behavior, the memory windows—defined as the difference between the threshold voltages in the forward and backward sweeps—were extracted from the cyclic transfer curves in Figure 2b and plotted as a function of the V_G_ sweep ranges, as shown in Figure 2c. The data reveal trends consistent with those observed under dark conditions: the memory window increases with increasing V_G_ sweep range and also increases with increasing shell thickness. These results further support the conclusion that the charge storage capability is enhanced in QDs with thicker shells, and that this behavior persists under both dark and illuminated environments.

It is well established that a clockwise hysteresis in the transfer curve of a transistor typically signifies hole trapping under negative gate bias V_G_ and electron trapping under positive V_G._^[^ ^8^, ^38^, ^39^ ^]^ However, a notable difference can be observed between Figure 2a (dark conditions) and Figure 2b (under illumination). In Figure 2a, the backward curves leftward shift along with the increasing V_G_ sweep range, which indicates a growing population of trapped holes resulting from increasingly negative V_G._^[^ ^8^, ^18^, ^40^ ^]^ Surprisingly, the forward sweeps also exhibit a leftward shift as the sweep range widens, which contradicts the typical expectation that a stronger positive V_G_ should promote electron trapping and thus cause a rightward shift.^[^ ^8^, ^18^, ^41^ ^]^ This anomaly suggests that during the sweeping process, the applied positive V_G_ is insufficient to fully neutralize the holes stored by the preceding negative V_G_ of the same magnitude. In other words, the number of electrons injected during the positive V_G_ phase is too low to compensate for the previously trapped holes, possibly due to a limited electron density or injection barrier at the QD interface. In contrast, under 405 nm illumination (Figure 2b), the transfer curves show a leftward shift in the backward sweeps and a rightward shift in the forward sweeps with increasing sweep range. This behavior indicates that both holes and electrons are effectively trapped under illumination, suggesting that photoexcitation generates sufficient electrons for storage in the QDs. To further verify the charge storage behavior, we directly monitored the dynamic response of source‐drain current (I_DS_) after applying a light pulse, as shown in Figure 2d. The light pulse induces electron trapping in the QDs, leading to an increase in I_DS_. Over time, as the stored electrons gradually relax, I_DS_ decays back to its baseline value. The results clearly show that as the ZnSe shell thickness increases, the decay becomes slower, indicating stronger charge retention and more effective charge‐holding ability in thicker‐shell QDs. These results clearly demonstrate that core–shell quantum dots with a type‐I energy band alignment possess effective charge storage capability, and that this capability exhibits a positive correlation with shell thickness.^[^ ^18^, ^32^ ^]^

The observed monotonic increase of memory window and slower current relaxation with thicker ZnSe shells is consistent with a type‐I alignment in which increasing shell thickness effectively enlarges the barrier for carrier escape from the InP core while suppressing interfacial recombination. This expectation is supported by theoretical considerations (Note S1, Supporting Information)^[^ ^42^ ^]^ and by experimental reports.^[^ ^18^, ^43^, ^44^ ^]^ Developing a full quantitative model that links shell thickness to barrier parameters for the specific InP/ZnSe QDs studied here would require extensive additional measurements beyond the present scope. Accordingly, we limit our analysis to these consistency checks.

**Figure 3:**
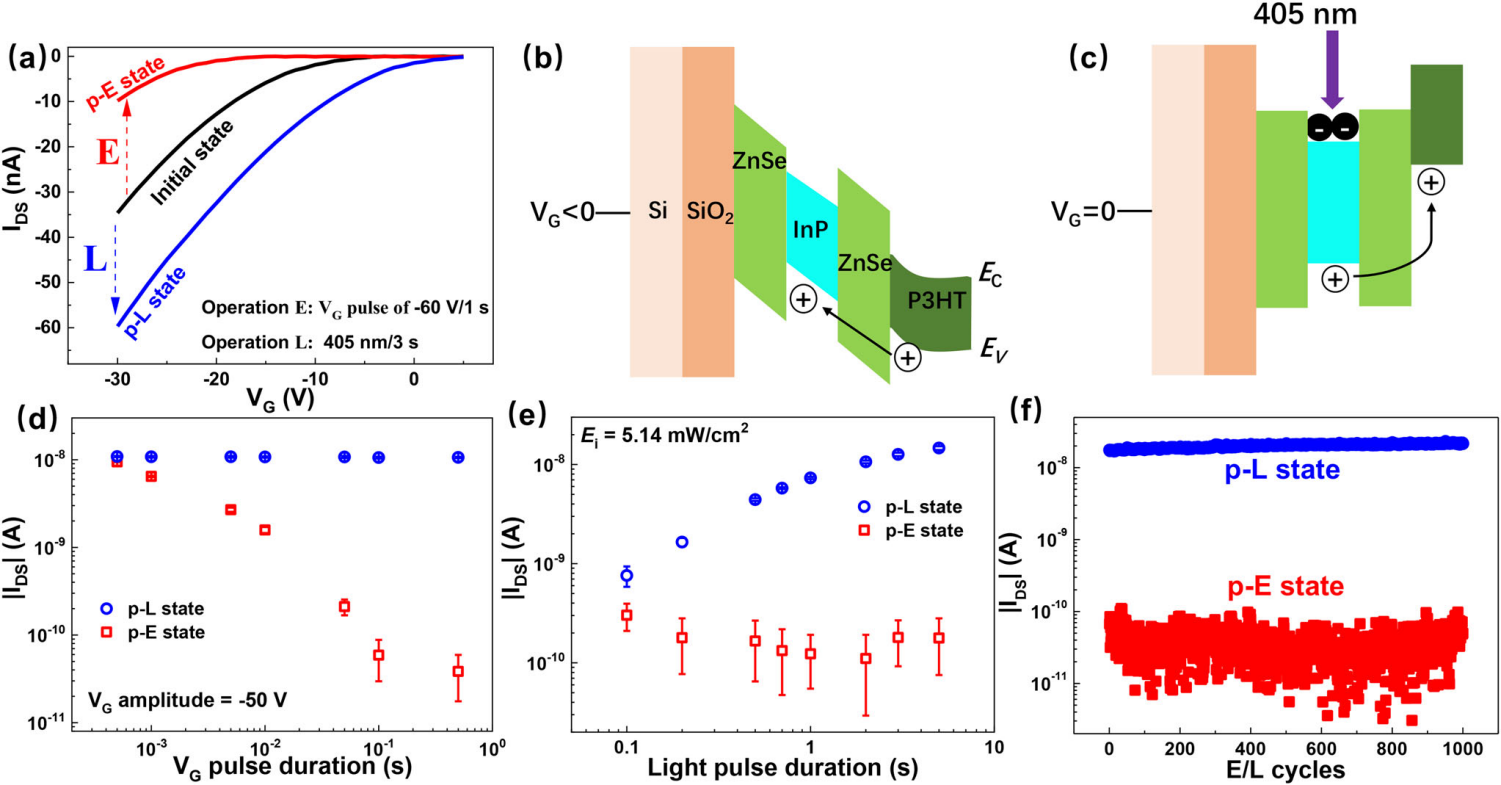
Impact of a V_G_ pulse of −60 V for 1 s (operation E) and 405 nm light illumination of 5.14 mW cm^−^^2^ for 3 s) (operation L) on the transfer characteristics of the InP/ZnSe‐thick‐device. V_DS_ = −30 V; Illustrations of holes storage process under negative V_G_ (b) and electron storage process under 405 nm light illumination (c). *E*_C_ indicates the conduction band, and *E*_V_ the valence band; d) Modulation of I_DS_ using operation E of −50 V V_G_ pulses with varying durations and operation L of 405 nm light pulses (5.14 mW cm−^2^, 2 s); e) Modulation of I_DS_ using operation E of V_G_ pulses (−50 V/0.1 s) and operation L of 405 nm light pulses with varying durations; f) Repetitive switch of the I_DS_ between p‐E and p‐L states separately induce by operation E of V_G_ pulse(60V/0.1 s) and operation L of 405 nm light illumination (5.14 mW cm^−2^, 3 s) for 1000 times. The I_DS_ in (d), (e), and (f) are all recorded at V_G_ = 0 V and V_DS_ = −30 V.

Based on the previously established observations that a negative V_G_ induces hole storage and that 405 nm light illumination induces electron storage in the QDs, the device can be reversibly modulated between two distinct states using electrical and optical stimuli. **Figure** **3**a shows the impact of a V_G_ pulse of −60 V for 1 s (denoted as operation E) and 405 nm light illumination of 5.14 mW/cm^2^ for 3 s) (denoted as operation L) on the transfer characteristics of the InP/ZnSe‐thick‐device. Starting from an initial state, operation E results in a leftward shift of the transfer curve to a post‐operation E (p‐E) state, while operation L induces a rightward shift to a post‐operation L (p‐L) state. The underlying mechanism is illustrated from a band structure perspective. It is widely accepted that InP/ZnSe core‐shell QDs have a type‐I band structure.^[^ ^30^ ^]^ The absorbance spectra of the InP/ZnSe‐thick QDs and P3HT are shown in Figure S1a (Supporting Information), with the corresponding Tauc plots provided in Figure S1b (Supporting Information).^[^ ^45^ ^]^ Based on the Tauc analysis, the optical band gaps of the InP core and the P3HT are estimated to be ≈2.1 and 2.0 eV, respectively. From literature values and empirical estimations, the conduction band (*E*_C_) and valence band (*E*_V_) of the InP are estimated to be −4.0 and −6.1 eV, respectively.^[^ ^8^, ^46^ ^]^ For P3HT, the *E*_C_ and *E*_V_ are estimated to be −3.0 and −5.0 eV, respectively.^[^ ^47^, ^48^ ^]^ Figure 3b shows that under a negative V_G_, a large number of holes accumulate in the *E*_V_ of the P3HT layer. This accumulation provides sufficient energy for holes in the P3HT to overcome the ZnSe shell barrier, enter the InP core, and become confined within the core due to the type‐I band alignment.^[^ ^8^, ^18^, ^49^ ^]^ In contrast, Figure 3c illustrates that under 405 nm light illumination, electrons and holes are generated within the InP core. Due to the favorable energy alignment of the *E*_V_, holes migrate from the QDs to the P3HT layer, leaving electrons trapped in the core, thereby modulating the device state.^[^ ^8^ ^]^ It is important to note that 405 nm falls within the strong absorption region of the QDs but lies in the weak absorption region of P3HT, as demonstrated by the absorption spectra in Figure S3a (Supporting Information). To validate this, we measured the dynamic I_DS_ response under 405 and 532 nm light pulses (both 4 s duration, same irradiance), as shown in Figure S3b (Supporting Information). The 532 nm light, which is strongly absorbed by P3HT but weakly by QDs (Figure S3a, Supporting Information), produced a much smaller change in I_DS_ than the 405 nm light. These results confirm that the QDs are the dominant contributors to the device's photoresponse.

The device can be reversibly switched between the p‐E and p‐L states, and the I_DS_ of the states can be tuned by adjusting the durations of the V_G_ and light pulses. Figure 3d shows the modulation of I_DS_ using operation E of −50 V V_G_ pulses with varying durations and operation L of 405 nm light pulses (5.14 mW cm^−2^, 2 s). The I_DS_ decreases with longer V_G_ pulse durations, indicating enhanced hole trapping. Notably, even a short V_G_ pulse of 1 ms is sufficient to induce a distinct state change. Similarly, Figure 3e presents the response to operation E of fixed V_G_ pulses (−50 V/0.1 s) and operation L of 405 nm light pulses with varying durations. The I_DS_ in the p‐L state increases with illumination time, reflecting increased electron trapping. Finally, Figure 3f demonstrates endurance performance, showing that the device can reliably switch between p‐L and p‐E states for over 1000 cycles, indicating excellent stability and repeatability.

### 2.2 Artificial Optoelectronic Synapse Application

**Figure 4:**
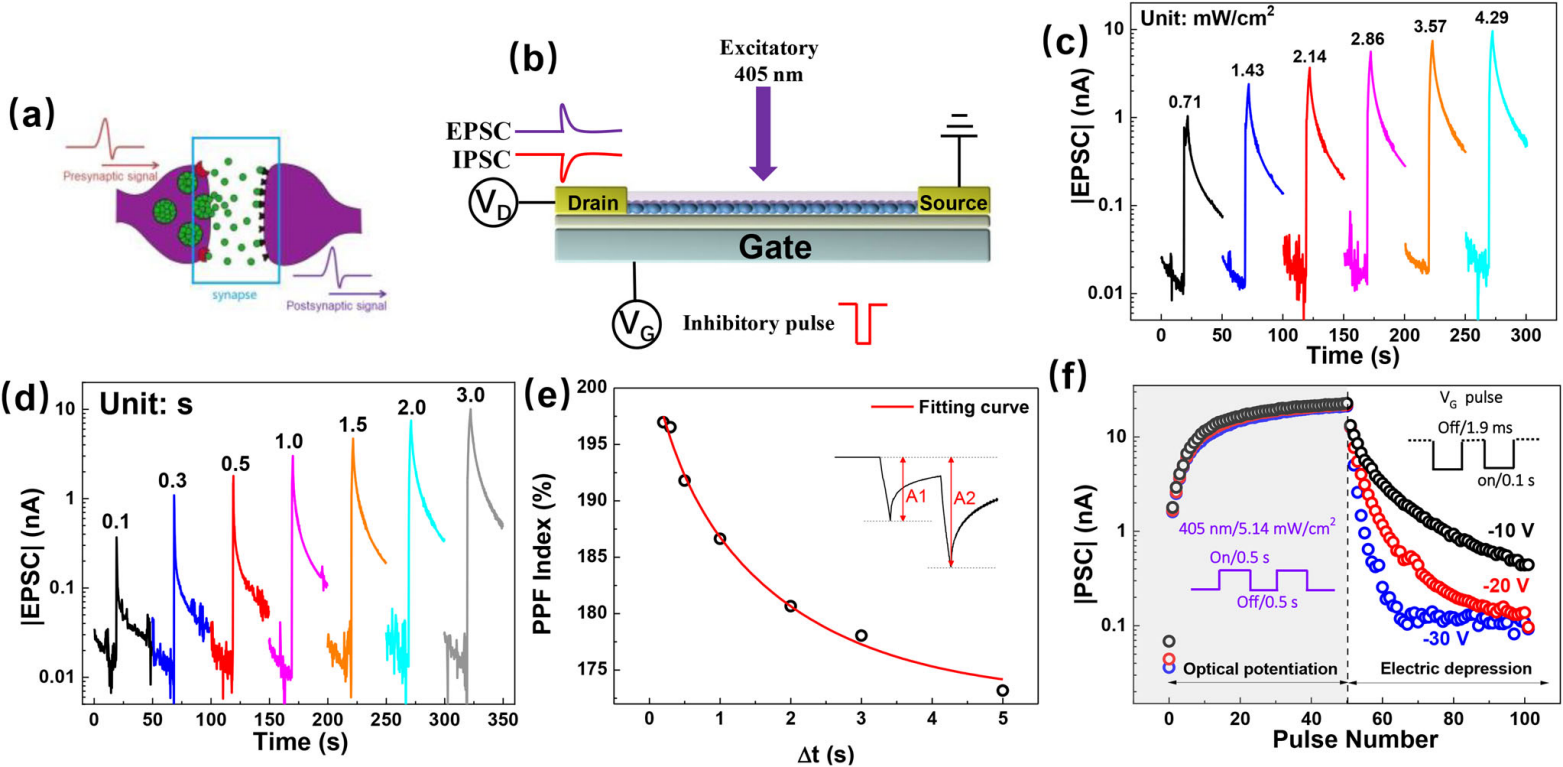
a) Working principle of a biological synapse; b) Analogous operation of the InP/ZnSe‐thick‐device as an artificial synapse; c) EPSC responses induced by light pulses with a fixed duration of 3 s and varying irradiance (c), and with a fixed irradiance of 5.14 mW cm^−^^2^ and varying duration (d); e) PPF index – Δt curve of the device. The inset illustrates the ESPC induced by two successive stimuli; f) Postsynaptic currents (PSC) potentiation‐depression cycles of the artificial synapse realized by optical and electric stimuli. The light irradiance in (d), (e), (f) is 5.14 mW cm^−^^2^.

The charge storage behavior of the QDs enables a state memory effect in the device, which can be harnessed to emulate biological synaptic functions. **Figure** **4**a illustrates the working principle of a biological synapse, where excitatory and inhibitory presynaptic spikes prompt the release of different neurotransmitters. These induce corresponding excitatory or inhibitory postsynaptic currents (EPSC and IPSC), modulating the synaptic connection strength, a process known as synaptic plasticity.^[^ ^14^ ^]^ The analogous operation of the InP/ZnSe‐thick‐device as an artificial synapse is shown in Figure 4b. Here, 405 nm light illumination and negative V_G_ pulses are used to mimic excitatory and inhibitory presynaptic stimuli, respectively. The drain current (I_DS_), measured at V_G_ = 0 V and V_DS_ = −30 V, serves as the postsynaptic response, representing either EPSC or IPSC depending on the input stimulus. Figure 4c,d presents the EPSC responses induced by sequences of light pulses. In Figure 4c, the pulse duration is fixed at 3 s while the irradiance increases. In Figure 4d, the irradiance is fixed while the pulse duration increases. In both cases, the EPSC amplitude increases with the strength of the stimulus, demonstrating the device's tunable synaptic weight and analog programmability. Figure 4d demonstrates that a light pulse of 0.1 s duration with an *E_i_* of 5.14 mW cm^−2^ is sufficient to act as a synaptic spike, producing a noticeable EPSC. The optoelectronic energy consumption per spike is estimated using the relation: *p*  = *E_i_*  × *A* × *t*, where *A* is the channel area, and *t* is the light pulse duration. Using this equation, we calculate a minimum *p* of ≈2.1 µJ. While this energy value and the response speed are higher than those reported in some cutting‐edge neuromorphic devices and biological synapses (typically < pJ energy and < µs response time),^[^ ^5^, ^6^, ^22^, ^50^, ^51^ ^]^ they still fall within the reasonable operating range of many mainstream artificial synaptic devices and systems.^[^ ^52^, ^53^, ^54^, ^55^ ^]^ These results demonstrate the feasibility of our platform for neuromorphic applications, while also indicating clear avenues for optimization.

To evaluate short‐term synaptic plasticity, the device was tested for paired‐pulse facilitation (PPF), a phenomenon where the second postsynaptic current (PSC, A_2_) exceeds the first (A_1_) when two identical presynaptic stimuli are applied in quick succession. PPF was emulated using two identical 405 nm light pulses (1 s duration), with the inter‐pulse interval (Δt) varying from 0.1 s to 5 s. The PPF index, defined as A_2_/A_1_, is plotted in Figure 4e as a function of Δt, showing a decay from 196% to 173% as Δt increases. The data fit well with a double exponential decay model:

(1) $$y=C_{0}+C_{1}e^{-\frac{\Delta t}{t_{1}}}+C_{2}e^{-\frac{\Delta t}{t_{2}}}$$

where C_1_ and C_2_ represent facilitation strengths, and t_1_ and t_2_ are decay time constants.^[^ ^56^ ^]^ The extracted values, t_1_ = 0.53 s and t_2_ = 0.75 s, correspond to the fast and slow decay processes, respectively.^[^ ^57^ ^]^ These two time constants can be interpreted as reflecting different relaxation dynamics of the trapped charge carriers following the first pulse. When the second pulse arrives shortly after the first (small Δt), the residual photogenerated charges have not fully decayed, leading to a stronger cumulative response. In contrast, at longer Δt, the system has more fully relaxed, so the second pulse yields a smaller facilitation effect. This behavior mimics short‐term synaptic facilitation in biological synapses, where the transient accumulation of Ca^2^⁺ ions at the presynaptic terminal increases neurotransmitter release probability for a second stimulus.^[^ ^58^ ^]^ This interpretation is conceptually aligned with models like those of Graupner and Brunel.^[^ ^59^ ^]^ Another important feature of synaptic plasticity is potentiation and depression, which correspond to the strengthening and weakening of synaptic weights. In the artificial synapses, potentiation is achieved using light pulses, while depression is triggered by negative V_G_ pulses. Figure 4f displays potentiation–depression cycles using 50 light pulses followed by 50 V_G_ pulses. The light pulses were applied at a frequency of 1 Hz, with a duration of 0.5 s. The V_G_ pulses were applied at 0.5 Hz, with a duration of 0.1 s, and varying magnitudes of −10, −20, and −30 V. The results show that stronger electrical pulses lead to faster depression, reflecting the controllable and bidirectional synaptic modulation of the device.

**Figure 5:**
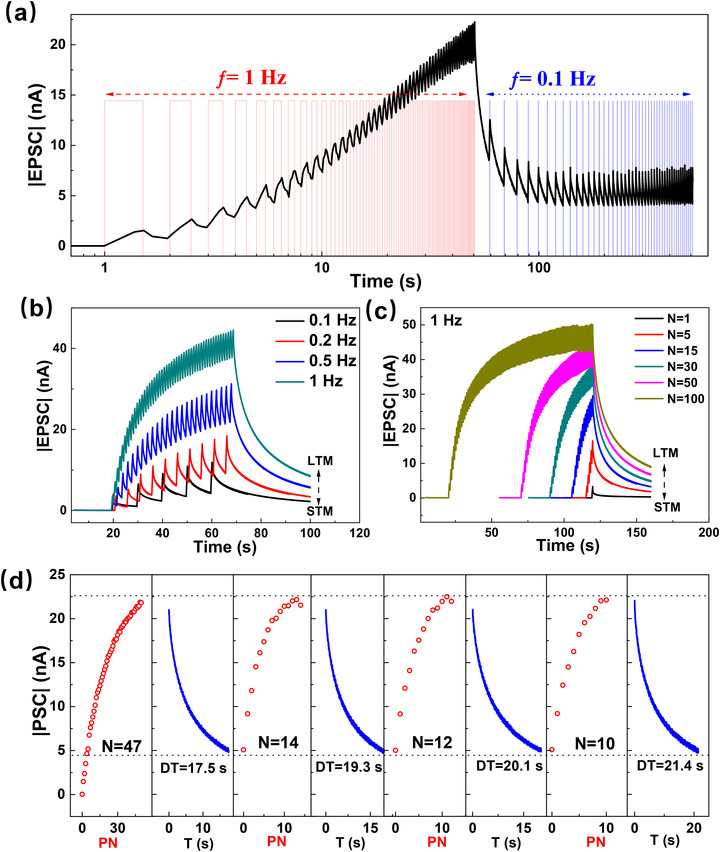
a) EPSC response by applying 405 nm light pulses at different frequencies; b) Evolution of EPSC by applying light pulses at different frequencies (0.1, 0.2, 0.5, and 1 Hz) over 50 s (b), or by applying light pulses with different pulse numbers (c); d) Evolution of EPSC by applying light pulses and then decay. This process repeats four times with different pulse numbers and decay durations. PN: pulse number; DT: decay time. The light pulse used in this figure has a duration of 0.5 s and an irradiance of 5.14 mW cm^−2^.

Spike‐rate‐dependent plasticity (SRDP) is a critical form of synaptic plasticity in biological neural systems, referring to changes in synaptic strength that depend on the frequency of presynaptic spiking.^[^ ^7^, ^60^ ^]^ This behavior is successfully emulated in our device using 405 nm light pulses as excitatory presynaptic stimuli. As shown in **Figure** **5**a, when light pulses are applied at a high frequency (1 Hz), the device exhibits synaptic potentiation, whereas at a low frequency (0.1 Hz), synaptic depression occurs. Both SRDP and paired‐pulse facilitation (PPF) are implicated in human cognitive behaviors, including learning and memory. Their successful reproduction in our device suggests that neuromorphic functionalities, such as memory formation and experience‐based learning, can be realized using light signals as input. Memory behavior in biological systems is typically categorized into short‐term memory (STM) and long‐term memory (LTM). STM lasts from seconds to minutes, whereas LTM can persist for hours, days, or even years. The process by which STM transitions into LTM is known as memory consolidation. Repeated learning, increased learning frequency, or rehearsal can facilitate this consolidation process.^[^ ^61^, ^62^ ^]^ Figure 5b shows the generation and decay of EPSC responses by applying light stimulus at 0.1, 0.2, 0.5, and 1 Hz over a 50‐s period. As the frequency increases, both the peak EPSC value and the retention time increase, closely resembling the transition from STM to LTM. It is worth noting that synapses exhibit metaplasticity, which means that the synapse's previous history of activity determines its current plasticity, including the STM‐LTM transition and SRDP.^[^ ^63^ ^]^ Before each measurement in Figure 5b, the device was depressed to a defined baseline with a negative V_G_ to ensure curve comparability and also to keep a constantly low potentiation threshold. While in Figure 5a, the 0.1 Hz train followed a prior 1 Hz potentiation sequence, which shifted the potentiation threshold to higher frequencies.^[^ ^60^ ^]^ This explains why a 0.1 Hz light pulse yields depression in Figure 5a but potentiation in Figure 5b. Metaplasticity is an inherent biological mechanism that prevents saturation of synaptic weights and maintains a balance between flexibility and stability in learning.^[^ ^64^ ^]^ Analogously, it can protect the optoelectronic synapses developed here from continuous potentiation or structural breakdown. Nevertheless, excessive metaplasticity may reduce the reproducibility of weight modulation. This effect could be alleviated by increasing the charge‐trapping capacity—such as by introducing a higher density of QDs or additional trap centers at the QD interface—to expand the modulation range and enhance stability. Figure 5c illustrates the effect of varying the number of light pulses at a fixed frequency of 1 Hz. As the number of pulses increases, the device exhibits stronger and more persistent EPSC responses, further demonstrating the STM‐to‐LTM transition.

A more sophisticated memory behavior—rehearsal‐induced consolidation—is demonstrated in Figure 5d, mimicking learning through experience.^[^ ^7^, ^65^ ^]^ Initially, 47 light pulses are applied at 1 Hz, inducing a strong EPSC. After cessation of the stimulus, the EPSC decays to a baseline level in 17.5 s. After that, only 14 light pulses are needed to recover the EPSC to the level before the decay. Then, the EPSC decays more slowly, taking 19.3 s to return to baseline. This cycle is repeated twice more, and it is observed that with each repetition, fewer pulses are required to restore the EPSC, and the decay time becomes longer, closely mimicking the biological rehearsal and memory reinforcement process.

### 2.3 Pavlov's Dog Experiment

**Figure 6:**
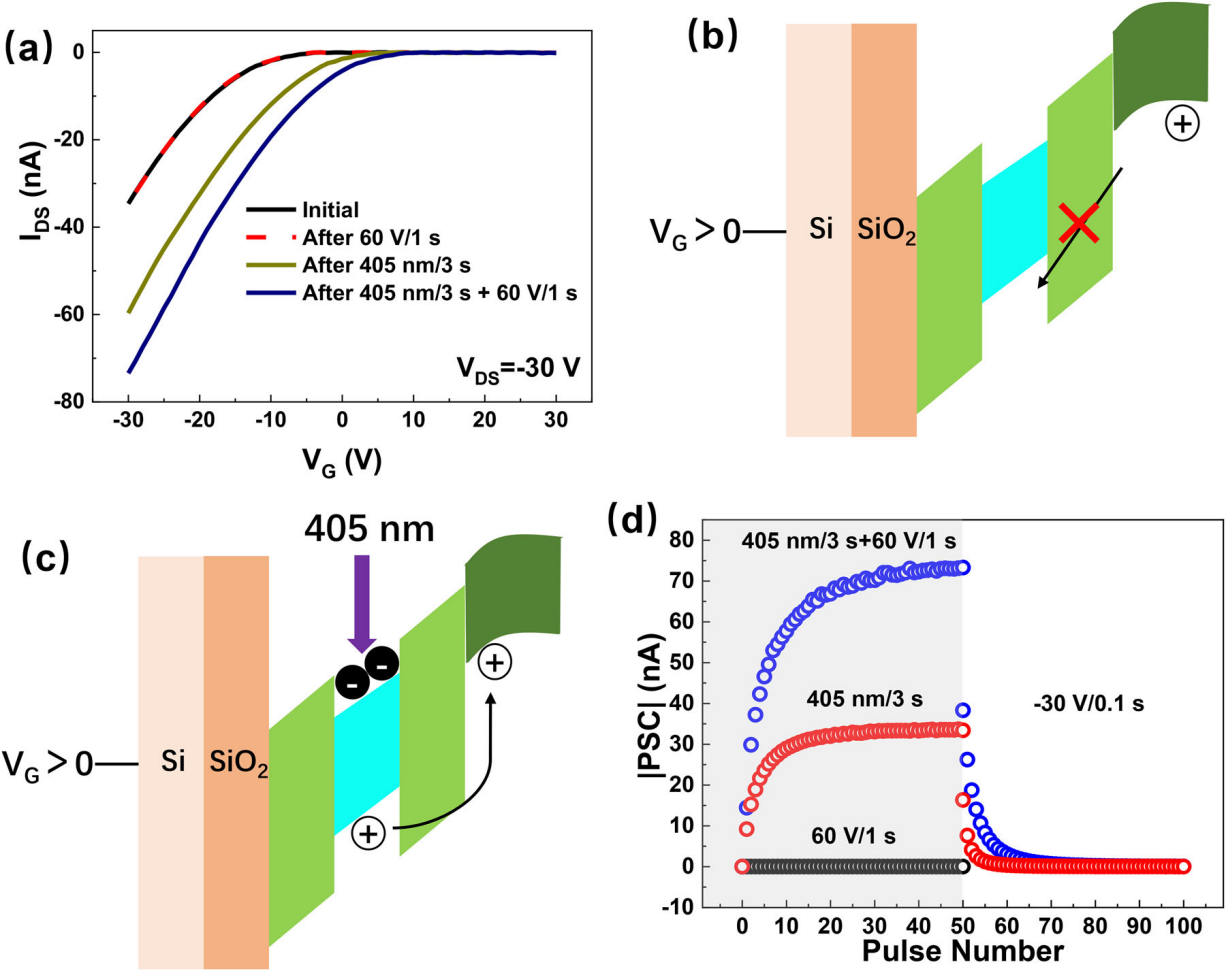
a) Transfer characteristics of the InP/ZnSe‐thick‐device after different stimuli; Energy band diagram under the dark condition (b) and under 405 nm illumination (c) with a positive V_G_ applied; d) Synaptic potentiation–depression cycles using three different potentiation stimuli—50 V_G_ pulses of 60 V/1 s, 50 light pulses of 405 nm/3 s, and 50 combined stimuli (405 nm/3 s+60 V/1 s)—with 50 common depression stimuli of −30 V/1 s. The irradiance of the 405 nm light in this figure is 5.14 mW cm^−2^.

It is noteworthy that the InP/ZnSe‐thick‐device exhibits inertness to positive V_G_ alone, but positive V_G_ significantly enhances the photoresponse of the device when combined with light illumination. Starting from the initial state, **Figure** **6**a presents the transfer characteristics recorded after different stimuli. A V_G_ pulse of 60 V and 1 s duration (60 V/1 s) results in no observable change in the transfer curve, indicating that positive V_G_ alone does not induce charge trapping. In contrast, a 405 nm light pulse of 3 s duration (405 nm/3 s) causes a rightward shift in the transfer curve, as expected from electron trapping induced by photoexcitation. Interestingly, when positive V_G_ and 405 nm light are applied simultaneously (405 nm/3 s+60 V/1 s), the transfer curve exhibits a more pronounced rightward shift compared to light illumination alone (405 nm/3 s), suggesting that the optical response of the device can be effectively enhanced by applying a positive gate bias. This indicates that photo‐induced electron trapping in the QDs can be synergistically enhanced by a positive V_G_. The physical mechanism is illustrated in Figure 6b,c, from the perspective of energy band diagrams. As shown in Figure 6b, under dark conditions, a positive V_G_ depletes the P3HT film, leading to the energy tilt of the system that is favorable for hole transfer from QDs to P3HT. Nevertheless, since there are no available charges in QDs, no charge transfer occurs. Under 405 nm light illumination (Figure 6c), electron–hole pairs are generated in the QDs. A positive V_G_ tilts the energy bands, promoting hole transfer from the QDs to P3HT, while electrons are retained in the InP core. This field‐assisted separation enhances charge trapping more effectively than illumination alone (as previously illustrated in Figure 3c). Figure 6d compares the synaptic potentiation–depression behaviors under three different potentiation stimuli: 50 V_G_ pulses of 60 V/1s, 50 light pulses of 405 nm/3 s, and 50 combined stimuli (405 nm/3 s+60 V/1 s), all followed by 50 depression stimuli of −30 V/0.1 s. The results clearly show that the combined stimulus leads to a larger synaptic weight, confirming the enhanced efficacy of field‐assisted photo‐induced charge trapping.

**Figure 7:**
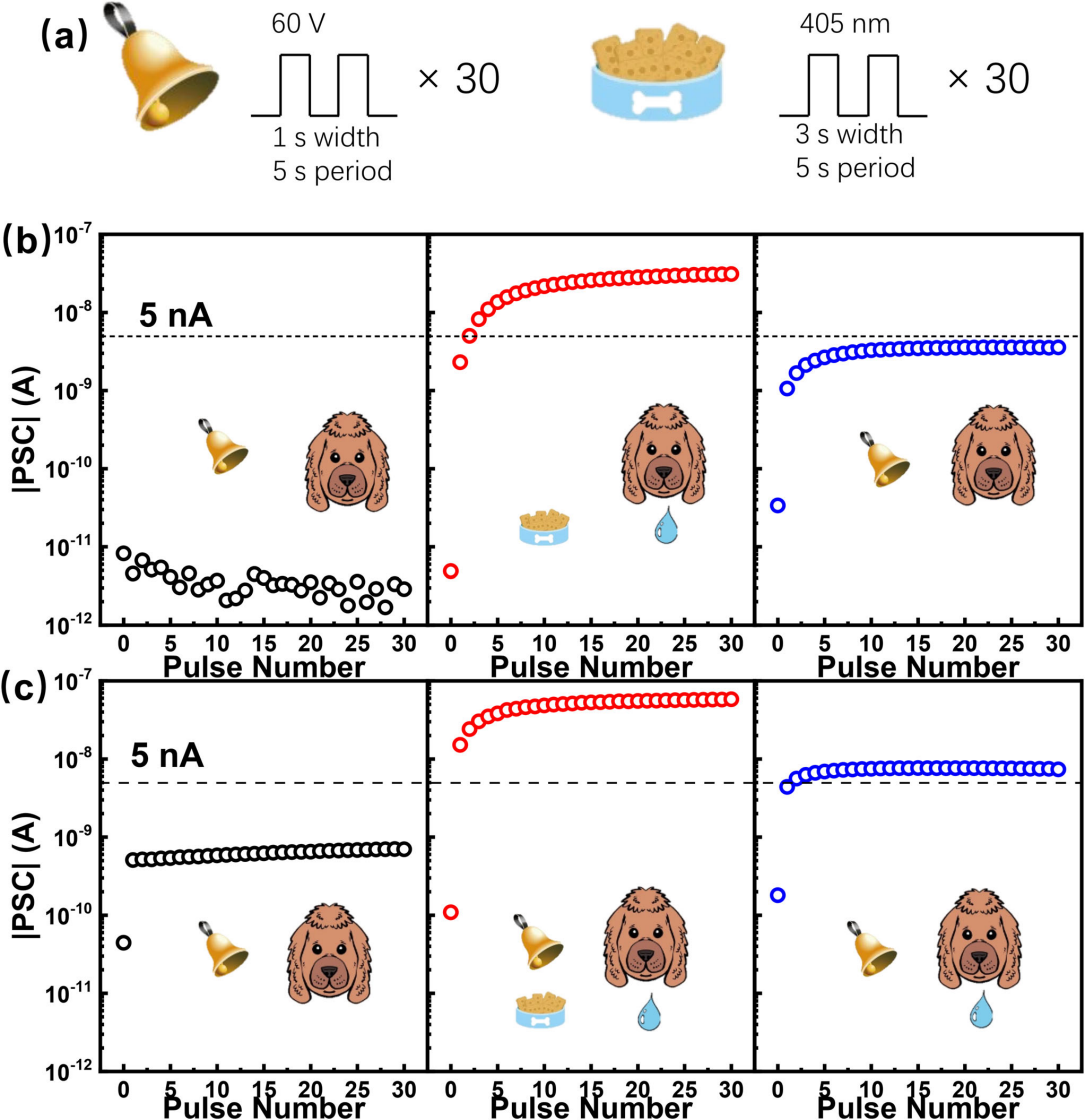
a) Schematic of the classical conditioning protocol; b) Response of the device to each stimulus solely; c) Building of the conditioned response. The stimuli are applied to the device successively from left to right in (b) and (c). The light irradiance in the figure is 5.14 mW cm^−2^.

Pavlov's dog experiment is a classical demonstration of associative learning, known as classical conditioning, in which a conditioned stimulus (e.g., a bell) becomes associated with an unconditioned stimulus (e.g., food) to elicit a learned response (e.g., salivation). In the context of neuromorphic electronics, such behavior reflects the capability for stimulus association and memory formation in hardware systems.^[^ ^23^ ^]^ Benefiting from the long‐term memory (LPM) features of the InP/ZnSe‐thick‐device, the gate field‐assisted photo‐induced charge trapping enables the emulation of classical conditioning behavior, as shown in **Figure** **7**. In this experiment, a train of positive V_G_ pulses (60 V/1 s, 0.2 Hz, 30 pulses) is defined as the conditioned stimulus (“bell ringing”), and a train of 405 nm light pulses (3 s duration, 0.2 Hz, 30 pulses) is used as the unconditioned stimulus (“food”), as illustrated in Figure 7a. A PSC threshold of 5 nA is defined to represent the “salivation” response in Pavlov's experiment.

As shown in Figure 7b, initially, the “dog” exhibits no response to the bell, i.e., the V_G_ pulse train alone produces no PSC. In contrast, the “food” stimulus (light pulses) induces a strong PSC response exceeding 5 nA, simulating unconditioned salivation. After this unpaired training process, the “bell ringing” alone is insufficient to induce a PSC beyond 5 nA. In contrast, Figure 7c shows that a combined stimulus (light + positive V_G_) during training leads to more charge trapping, increasing the saturation level of the PSC. After the paired training, the “bell ringing” stimulus (V_G_ pulses) alone is sufficient to induce a PSC above the 5 nA threshold, effectively emulating the conditioned salivation response—thereby demonstrating that classical conditioning has been established in the device. Please note that i) In both Figure 7b,c, the three operations are conducted sequentially. Between each operation, the device is reset to its baseline state by applying a negative gate voltage pulse (−60 V for 10 s). ii) After either the “food” stimulus or the “food + bell” combination, subsequent exposure to the “bell ringing” (positive V_G_ pulse) alone produces a PSC response, unlike the fresh unconditioned state. Therefore, we speculate the reason underlying this process as follows: Both the “food” alone and “food + bell ringing” stimuli lead to electron storage in the core of the quantum dots (Figure S4a,d, Supporting Information). Upon application of the recovery pulse (−60 V/10 s), the stored electrons are released into the P3HT layer, where they can be transiently trapped in localized states (Figure S4b,e, Supporting Information). When a positive V_G_ pulse is subsequently applied, these trapped electrons are reinjected into the QDs, giving rise to a measurable PSC (Figure S4c,f, Supporting Information). The key difference lies in the electron density generated during conditioning: the combined “food + bell ringing” stimulus results in a higher population of stored electrons, leading to more extensive trapping in P3HT and ultimately a larger reinjected electron population—enough to produce a PSC above the 5 nA threshold. In contrast, the “food” stimulus alone does not generate sufficient electron density to achieve this threshold upon V_G_ stimulation. For comparison, Table S1 (Supporting Information) summarizes key traits of recently reported artificial optoelectronic synapses employing quantum dots as charge‐trapping centers. The present InP/ZnSe QD‐based synapses achieve diverse and stable neuromorphic functionalities using moderate pre‐synaptic spike intensities, without the need for a blocking dielectric layer. These results demonstrate the feasibility and practical promise of type‐I core–shell QDs for energy‐efficient and environmentally benign neuromorphic optoelectronic devices.

## 3 Conclusion

In summary, we have demonstrated that InP/ZnSe core–shell quantum dots (QDs) with a type‐I band alignment exhibit enhanced charge storage capabilities that are positively correlated with shell thickness. By integrating these QDs into P3HT‐based thin‐film transistors, we constructed a neuromorphic optoelectronic device capable of bidirectional electrical and optical modulation. The charge‐trapping behavior within the QDs enabled the device to emulate a broad range of synaptic functions, including frequency‐dependent potentiation and depression, paired‐pulse facilitation, spike‐rate‐dependent plasticity, and short‐ to long‐term memory transitions. Notably, the field‐assisted photoinduced charge trapping was leveraged to emulate Pavlovian associative learning, highlighting the device's capacity for complex learning behavior. These results not only establish a promising platform for neuromorphic visual systems but also provide new insights into charge‐dynamic engineering in core–shell colloidal QDs for brain‐inspired hardware.

## 4 Experimental Section

### Materials

Regioregular Poly(3‐hexylthiophene‐2,5‐diyl) (P3HT, regioregularity≥90%, molecular weight: 50 000–75 000), Indium acetate (In(Ac)_3_,99.99%), palmitic acid (PA, 99%), and selenium (Se,100mesh, 99.99%) were purchased from Sigma‐Aldrich. Zinc stearate (Zn(St)_2_, ZnO 12.5–14%), 1‐octadecene (ODE, 90%) were purchased from Alfa Aesar. Tris(trimethylsily)phosphine ((TMS)_3_P, 10 wt% in hexane, >98%) and trioctylphosphine (TOP, 97%) were purchased from Strem. (TMS)_3_P was distilled prior to use and stored in a nitrogen‐filled glovebox.

### Synthesis of InP QDs

In(Ac)_3_ (0.4 mmol) and PA (1.2 mmol) were mixed with 10.0 mL of ODE in a 50 mL three‐neck flask. The solution was degassed at 120 °C under vacuum for 1 h. Then, the flask was refilled with N_2_ and heated to 280 °C for 10 min. Then, 0.2 mmol of (TMS)_3_P (dissolved in 1 mL TOP) was quickly injected into the flask. The temperature was decreased to 270 °C and maintained for 40 min to prepare the InP core. The obtained InP QDs were washed for two times with toluene and ethanol. Finally, InP QDs were re‐dispersed in 2.0 mL of toluene and stored in the glove box.

### Synthesis of InP/ZnSe QDs

Into the InP QDs solution before washing, 0.32 mmol of ZnSt_2_ in 2.0 mL of ODE was injected. After 10 min, 0.26 mmol of TOP‐Se was injected and the temperature was maintained at 270 °C for 15 min. The obtained QDs were washed for two times with toluene and ethanol. The residual was mixed with 0.92 mmol ZnSt_2_ and 10 mL TOA in a 50 mL three‐neck flask. The mixture was degassed at 80 °C. Then the solution was heated to 340 °C, followed by dropwise injection of 0.91 mmol TOP‐Se via syringe pump over 30 min to obtain InP/ZnSe‐thin QDs. For synthesizing InP/ZnSe‐thick QDs, the amounts of ZnSt_2_ and TOP‐Se were 6.55 mmol and 6.50 mmol, respectively, with corresponding reaction times of 90 min. The obtained InP/ZnSe QDs were washed for two times with toluene and ethanol. Finally, InP/ZnSe QDs were re‐dispersed in 2.0 mL of toluene and stored in the glove box.

### Device Fabrications

Si/SiO_2_ wafer with heavy p‐doping Si and SiO_2_ of 300 nm thickness, was used as the substrate. The p‐doping Si was used as the gate electrode in the device measurement. QD films were deposited by spin‐coating the QD solutions at 2000 rpm for 60 s. The QD films were annealed at 60 °C for 1 h. Then, P3HT films were formed by spin‐coating P3HT solution in toluene of 5 mg mL^−1^ atop the QD films. After an annealing of 110 °C for 1 h, an Au source/drain electrode with a thickness of ≈50 nm was thermally evaporated onto the P3HT films through a shadow mask defining a channel with a width/length of 2/0.2 mm. All the device fabrication was carried out in a glovebox filled with nitrogen.

### Characterization

Transmission electron microscopy (TEM) images were acquired on an FEI Titan Cubed Themis G201 double spherical aberration‐corrected transmission electron microscope with an acceleration voltage of 300 kV. Absorbance spectra were recorded using a GBC Cintra2020 spectrometer with tunable wavelengths. Photoluminescence (PL) spectra were obtained on Spectrofluorometer FS5 (Edinburgh Instruments). The electrical measurement of the devices was conducted using a probe station combined with a semiconductor analyzer (4200 SCS, Keithley). A total of 405 nm (MDL‐III‐405‐50 mW) and 532 nm (MGL‐III‐532‐50 mW) continuous lasers with adjustable power and modulation frequency were employed as light sources for optoelectronic measurement. The lasers are delivered through an optical fiber mounted with a collimator. The output laser spots have a circular shape with a large size covering the whole chip. All the performance measurements were carried out in a glovebox filled with nitrogen.

## Conflict of Interest

The authors declare no conflict of interest.

## Supporting information



Supporting Information

## Data Availability

The data that support the findings of this study are available on request from the corresponding author. The data are not publicly available due to privacy or ethical restrictions.

## References

[1] Q. Wan , C. Wan , H. Wu , Y. Yang , X. Huang , P. Zhou , L. Chen , T.‐Y. Wang , Y. Li , K.‐H. Xue , Y.‐H. He , X.‐S. Miao , X. Li , C. Xie , H. Chen , Z. Song , H. Wang , Y. Hao , J. Zhang , J. Huang , Z. Y. Ren , L. Q. Zhu , J. Du , C. Ge , Y. Liu , G. Ding , Y. Zhou , S.‐T. Han , G. Wang , X. Yu , et al., Neuromorphic Computing and Engineering 2022, 2, 042501.

[2] F. Liao , F. Zhou , Y. Chai , J. Semicond. 2021, 42, 013105.

[3] Z. Wang , S. Joshi , S. E. Savel'ev , H. Jiang , R. Midya , P. Lin , M. Hu , N. Ge , J. P. Strachan , Z. Li , Q. Wu , M. Barnell , G.‐L. Li , H. L. Xin , R. S. Williams , Q. Xia , J. J. Yang , Nat. Mater. 2016, 16, 101. 27669052 10.1038/nmat4756

[4] M.‐K. Kim , Y. Park , I.‐J. Kim , J.‐S. Lee , iScience 2020, 23, 101846. 33319174 10.1016/j.isci.2020.101846 PMC7725950

[5] S. Dai , Y. Zhao , Y. Wang , J. Zhang , L. Fang , S. Jin , Y. Shao , J. Huang , Adv. Funct. Mater. 2019, 29, 1903700.

[6] X. Wang , Y. X. Ran , X. Q. Li , X. S. Qin , W. L. Lu , Y. W. Zhu , G. H. Lu , Mater. Horiz. 2023, 10, 3269. 37312536 10.1039/d3mh00216k

[7] Z. Wen , S. Wang , F. Yi , D. Zheng , C. Yan , Z. Sun , ACS Appl. Mater. Interfaces 2023, 15, 55916. 37984451 10.1021/acsami.3c06590

[8] H. hu , G. Wen , J. Wen , L. B. Huang , M. Zhao , H. Wu , Z. Sun , Adv. Sci. 2021, 8, 2100513. 10.1002/advs.202100513 PMC8373160 34174170

[9] H.‐L. Loi , T. Wang , D. Liu , J. Cao , J. Zhuang , Z. Zhao , Y. Xu , M. G. Li , L. Li , T. Zhai , F. Yan , Adv. Funct. Mater. 2025, 35, 2422267.

[10] K. Tran , H. Wang , M. Pieters , M. A. Loi , Advanced Intelligent Systems 2023, 5, 2300218.

[11] W. C. Yang , E. Ercan , Y. C. Lin , W. C. Chen , Y. Watanabe , K. Nakabayashi , B. H. Lin , C. T. Lo , H. Mori , W. C. Chen , Adv. Opt. Mater. 2022, 11, 2202110.

[12] Y. Xu , Y. Shi , C. Qian , P. Xie , C. Jin , X. Shi , G. Zhang , W. Liu , C. Wan , J. C. Ho , J. Sun , J. Yang , Nano Lett. 2023, 23, 5264. 37229610 10.1021/acs.nanolett.3c01291

[13] F. Guo , M. Song , M. C. Wong , R. Ding , W. F. Io , S. Y. Pang , W. Jie , J. Hao , Adv. Funct. Mater. 2021, 32, 2108014.

[14] Z. Lv , Y. Wang , J. Chen , J. Wang , Y. Zhou , S.‐T. Han , Chem. Rev. 2020, 120, 3941. 32202419 10.1021/acs.chemrev.9b00730

[15] F. P. García de Arquer , D. V. Talapin , V. I. Klimov , Y. Arakawa , M. Bayer , E. H. Sargent , Science 2021, 373, aaz8541. 10.1126/science.aaz8541 34353926

[16] Z. Sun , J. Li , C. Liu , S. Yang , F. Yan , Nano Lett. 2020, 21, 723. 33373246 10.1021/acs.nanolett.0c04370

[17] K. Liang , R. Wang , B. Huo , H. Ren , D. Li , Y. Wang , Y. Tang , Y. Chen , C. Song , F. Li , B. Ji , H. Wang , B. Zhu , ACS Nano 2022, 16, 8651. 35451308 10.1021/acsnano.2c00439

[18] C. Yan , J. Wen , P. Lin , Z. Sun , Small 2018, 15, 1804156. 10.1002/smll.201804156 30480357

[19] Z.‐P. Wang , Y. Wang , J. Yu , J.‐Q. Yang , Y. Zhou , J.‐Y. Mao , R. Wang , X. Zhao , W. Zheng , S.‐T. Han , Nano Lett. 2020, 20, 5562. 32579373 10.1021/acs.nanolett.0c02227

[20] J. Wang , Z. Lv , X. Xing , X. Li , Y. Wang , M. Chen , G. Pang , F. Qian , Y. Zhou , S. T. Han , Adv. Funct. Mater. 2020, 30, 1909114.

[21] J. Y. Zhang , Z. Y. Guo , T. R. Sun , P. Guo , X. Liu , H. Y. Gao , S. L. Dai , L. Z. Xiong , J. Huang , SmartMat 2023, 14.

[22] J. Zhang , P. Guo , Z. Guo , L. Li , T. Sun , D. Liu , L. Tian , G. Zu , L. Xiong , J. Zhang , J. Huang , Adv. Funct. Mater. 2023, 33, 2302885.

[23] J.‐L. Meng , T.‐Y. Wang , L. Chen , Q.‐Q. Sun , H. Zhu , L. Ji , S.‐J. Ding , W.‐Z. Bao , P. Zhou , D. W. Zhang , Nano Energy 2021, 83, 105815.

[24] L. Li , X.‐L. Wang , J. Pei , W.‐J. Liu , X. Wu , D. W. Zhang , S.‐J. Ding , Sci. China Mater. 2020, 64, 1219.

[25] Z. Lv , M. Chen , F. Qian , V. A. L. Roy , W. Ye , D. She , Y. Wang , Z. X. Xu , Y. Zhou , S. T. Han , Adv. Funct. Mater. 2019, 29, 1902374.

[26] Y. Wang , Z. Lv , J. Chen , Z. Wang , Y. Zhou , L. Zhou , X. Chen , S. T. Han , Adv. Mater. 2018, 30, 1802883. 10.1002/adma.201802883 30063261

[27] A. Marent , M. Geller , A. Schliwa , D. Feise , K. Pötschke , D. Bimberg , N. Akçay , N. Öncan , Appl. Phys. Lett. 2007, 91, 243109.

[28] M. Geller , A. Marent , T. Nowozin , D. Bimberg , N. Akçay , N. Öncan , Appl. Phys. Lett. 2008, 92.

[29] W. A. Tisdale , K. J. Williams , B. A. Timp , D. J. Norris , E. S. Aydil , X. Y. Zhu , Science 2010, 328, 1543. 20558714 10.1126/science.1185509

[30] E. Jang , Y. Kim , Y.‐H. Won , H. Jang , S.‐M. Choi , ACS Energy Lett. 2020, 5, 1316.

[31] Y.‐H. Won , O. Cho , T. Kim , D.‐Y. Chung , T. Kim , H. Chung , H. Jang , J. Lee , D. Kim , E. Jang , Nature 2019, 575, 634. 31776489 10.1038/s41586-019-1771-5

[32] M. D. Tessier , D. Dupont , K. De Nolf , J. De Roo , Z. Hens , Chem. Mater. 2015, 27, 4893.

[33] C. d. M. Donegá , Chem. Soc. Rev. 2011, 40, 1512.20972490

[34] K. R. Reid , J. R. McBride , N. J. Freymeyer , L. B. Thal , S. J. Rosenthal , Nano Lett. 2018, 18, 709. 29282985 10.1021/acs.nanolett.7b03703 PMC6163126

[35] L. Li , P. Reiss , J. Am. Chem. Soc. 2008, 130, 11588. 18686956 10.1021/ja803687e

[36] K. Wang , H. Ling , Y. Bao , M. Yang , Y. Yang , M. Hussain , H. Wang , L. Zhang , L. Xie , M. Yi , W. Huang , X. Xie , J. Zhu , Adv. Mater. 2018, 30, 1800595. 10.1002/adma.201800595 29782682

[37] Y. J. Jeong , D.‐J. Yun , S. H. Noh , C. E. Park , J. Jang , ACS Nano 2018, 12, 7701. 30024727 10.1021/acsnano.8b01413

[38] P.‐F. Wang , X. Lin , L. Liu , Q.‐Q. Sun , P. Zhou , X.‐Y. Liu , W. Liu , Y. Gong , D. W. Zhang , Science 2013, 341, 640. 23929978 10.1126/science.1240961

[39] S. Wang , C. He , J. Tang , X. Lu , C. Shen , H. Yu , L. Du , J. Li , R. Yang , D. Shi , G. Zhang , Adv. Electron. Mater. 2019, 5, 1800726.

[40] M. Yi , M. Xie , Y. Shao , W. Li , H. Ling , L. Xie , T. Yang , Q. Fan , J. Zhu , W. Huang , J. Mater. Chem. C 2015, 3, 5220.

[41] J. Wen , H. Hu , G. Wen , S. Wang , Z. Sun , S. Ye , J. Phys. D: Appl. Phys. 2021, 54, 114002.

[42] M. G. Chapline , S. X. Wang , J. Appl. Phys. 2007, 101.

[43] J. Vela , H. Htoon , Y. Chen , Y.‐S. Park , Y. Ghosh , P. M. Goodwin , J. H. Werner , N. P. Wells , J. L. Casson , J. A. Hollingsworth , Journal of Biophotonics 2010, 3, 706. 20626004 10.1002/jbio.201000058 PMC2943988

[44] J. Hollingsworth , J. Vela , Y. Chen , H. Htoon , V. Klimov , A. Casson , in Colloidal Quantum Dots for Biomedical Applications IV, Vol. 7189, SPIE, San Jose, California, United States 2009, 718904. 10.1117/12.809678 PMC3146174 21804930

[45] P. Makuła , M. Pacia , W. Macyk , J. Phys. Chem. Lett. 2018, 9, 6814. 30990726 10.1021/acs.jpclett.8b02892

[46] H. Fu , A. Zunger , Phys. Rev. B 1997, 56, 1496.

[47] M. A. Ansari , S. Mohiuddin , F. Kandemirli , M. I. Malik , RSC Adv. 2018, 8, 8319. 35541991 10.1039/c8ra00555a PMC9078512

[48] Z. Sun , J. Li , F. Yan , J. Mater. Chem. 2012, 22, 21673.

[49] Y.‐C. Chen , C.‐Y. Huang , H.‐C. Yu , Y.‐K. Su , J. Appl. Phys. 2012, 112, 034518.

[50] T. Liu , Z. Yuan , L. Wang , C. Shan , Q. Zhang , H. Chen , H. Wang , W. Wu , L. Huang , Y. Chai , X. Meng , Nat. Commun. 2025, 16, 4261. 40335551 10.1038/s41467-025-59624-2 PMC12059062

[51] C. Liu , H. Chen , S. Wang , Q. Liu , Y.‐G. Jiang , D. W. Zhang , M. Liu , P. Zhou , Nat. Nanotechnol. 2020, 15, 545. 32647168 10.1038/s41565-020-0724-3

[52] Z. Wang , L. Wang , M. Nagai , L. Xie , M. Yi , W. Huang , Adv. Electron. Mater. 2017, 3, 1600510.

[53] C. Liu , C. Gao , W. Huang , M. Lian , C. Xu , H. Chen , T. Guo , W. Hu , Sci. China Mater. 2024, 67, 1500.

[54] F. Alibart , S. Pleutin , D. Guérin , C. Novembre , S. Lenfant , K. Lmimouni , C. Gamrat , D. Vuillaume , Adv. Funct. Mater. 2010, 20, 330.

[55] Y. Wang , W. Shan , H. Li , Y. Zhong , S. Wustoni , J. Uribe , T. Chang , V. E. Musteata , T. C. H. Castillo , W. Yue , H. Ling , N. El‐Atab , S. Inal , Nat. Commun. 2025, 16, 1615. 39948373 10.1038/s41467-025-56814-w PMC11825661

[56] T. Ahmed , S. Kuriakose , S. Abbas , M. J. S. Spencer , M. A. Rahman , M. Tahir , Y. Lu , P. Sonar , V. Bansal , M. Bhaskaran , S. Sriram , S. Walia , Adv. Funct. Mater. 2019, 29, 1901991.

[57] C. Du , W. Ma , T. Chang , P. Sheridan , W. D. Lu , Adv. Funct. Mater. 2015, 25, 4290.

[58] S. L. Jackman , W. G. Regehr , Neuron 2017, 94, 447. 28472650 10.1016/j.neuron.2017.02.047 PMC5865607

[59] M. Graupner , N. Brunel , Proc. Natl. Acad. Sci. USA 2012, 109, 3991. 22357758 10.1073/pnas.1109359109 PMC3309784

[60] Z. Y. Ren , L. Q. Zhu , Y. B. Guo , T. Y. Long , F. Yu , H. Xiao , H. L. Lu , ACS Appl. Mater. Interfaces 2020, 12, 7833. 31961648 10.1021/acsami.9b22369

[61] T. Ohno , T. Hasegawa , T. Tsuruoka , K. Terabe , J. K. Gimzewski , M. Aono , Nat. Mater. 2011, 10, 591. 21706012 10.1038/nmat3054

[62] A. Baddeley , Working Memory, Thought, and Action, Oxford University Press, Oxford, England 2007.

[63] W. C. Abraham , M. F. Bear , Trends Neurosci. 1996, 19, 126. 8658594 10.1016/s0166-2236(96)80018-x

[64] W. C. Abraham , Nat. Rev. Neurosci. 2008, 9, 387. 18401345 10.1038/nrn2356

[65] S. Ge , F. Huang , J. He , Z. Xu , Z. Sun , X. Han , C. Wang , L. B. Huang , C. Pan , Adv. Opt. Mater. 2022, 10, 2200409.

