# Supplementary material for: Controllable Charge Storability in InP/ZnSe Core/Shell Quantum Dots toward Bioinspired Optical Synaptic Application
Source: Adv Sci (Weinh). 2025 Nov 5;13(5):e11714. doi: 10.1002/advs.202511714 (PMC12850371; doi:10.1002/advs.202511714)
Supplement: Supplementary file 1 — Supporting Information [file ADVS-13-e11714-s001.pdf]

Supporting Information

Controllable Charge Storability in InP/ZnSe Core/Shell Quantum Dots Towards Bioinspired Optical Synaptic Application

Guohao Wen, Bingbing Huo, Dingting Zheng, Xiang Zheng, Guanlin Ke, Zhiguo Chi, Honglei Wu, Botao Ji, Zhenhua Sun**

Note 1:

For a type-I core–shell QD, carrier escape from the core across the shell can be approximated by a tunneling/thermionic process. In a simple 1D picture, the tunneling factor scales as

$$P \propto e^{(-2kd)}, \qquad k = \sqrt{\frac{2m^*}{\hbar^2}(\varphi_b - E)}$$

where d is shell thickness, m^* the carrier effective mass in the shell, φ_b an effective barrier, and E the carrier energy. The associated escape time t increases approximately as

$$t \propto t_0 e^{(2kd)}$$

so thicker shells (larger d) yield longer retention.

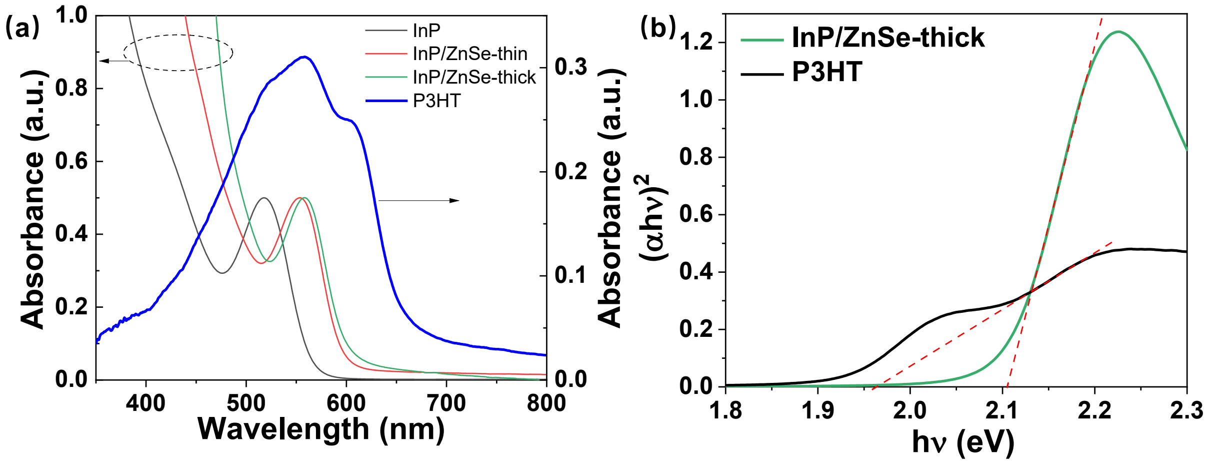

Figure S1. (a) Absorbance spectra of three types of QDs and the P3HT film; (b) The Tauc plots of InP/ZnSe-thick QDs and P3HT film.

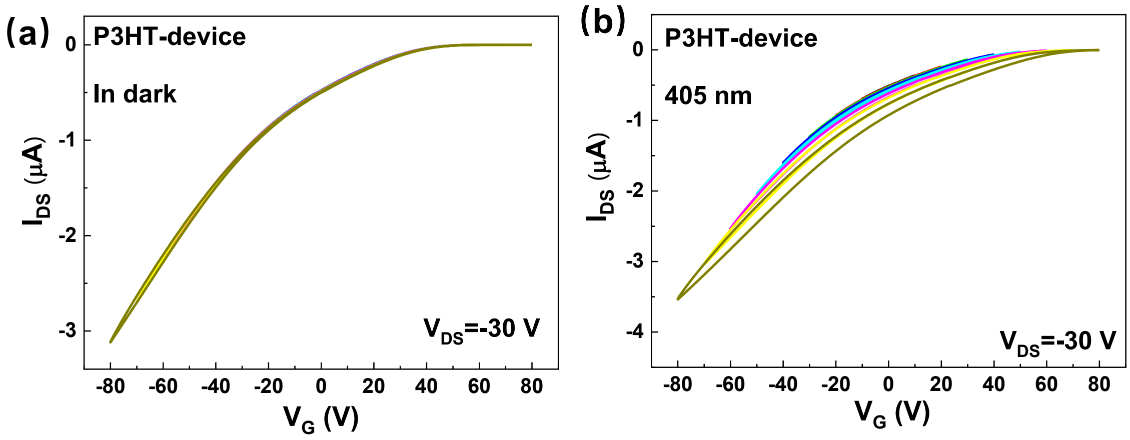

Figure S2. Cyclic transfer curves of the pristine P3HT-device measured with varying V_G and V_{DS}=-30 V in the dark(a) and under 405 nm light illumination(b).

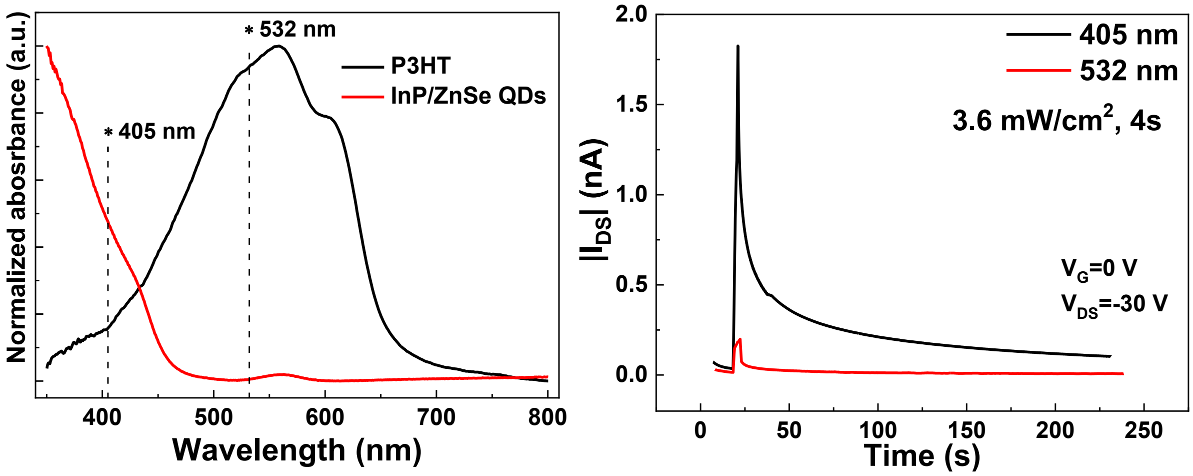

Figure S3. (a) Absorbance spectra of the QDs solution and the P3HT film; (b) Temperal I_{DS} response of the InP/ZnSe-thick-device to 405 nm and 532 nm lights with identical irradiance and duration.

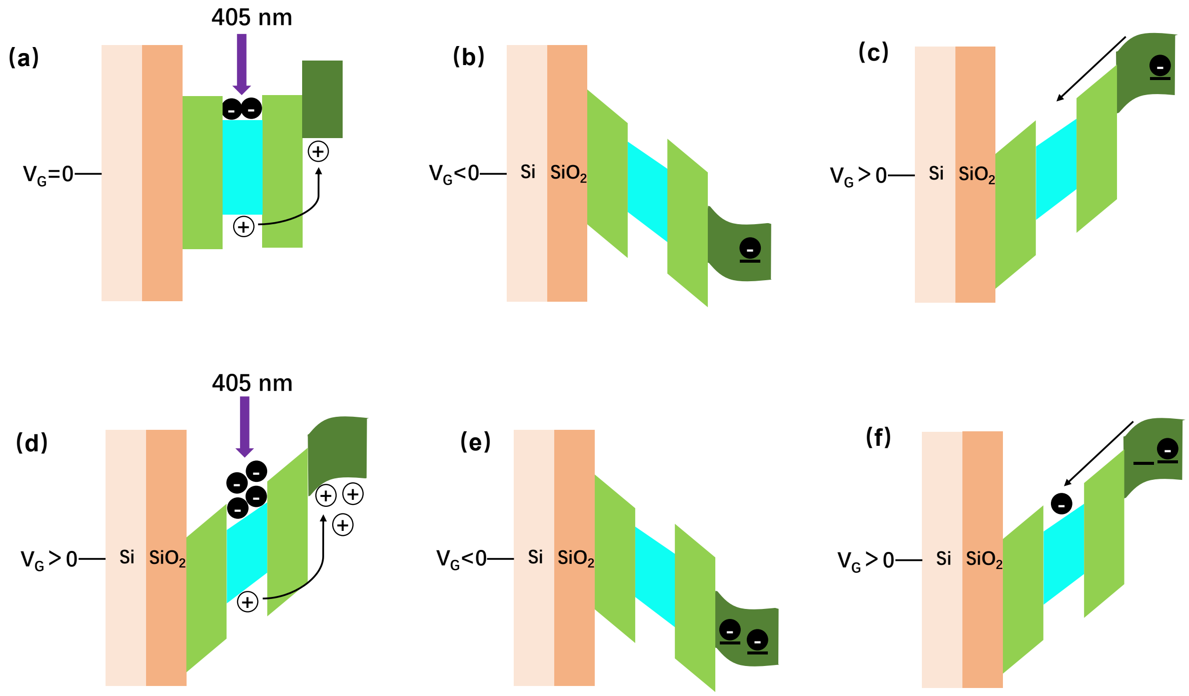

Figure S4. Illustrations of the associative learning process of the device. (a) Electron storage induced by light alone; (b) Relase of the stored electrons by a positive V_G; (c) Electron storage induced by a positive V_G. The process happens succesively from (a) to (c); (d) Electron storage induced by the combination of light and positive V_G; (b) Relase of the stored electrons by a negative V_G; (c) Electron storage induced by a positive V_G. The process happens succesively from (d) to (f).

Table S1. Summary of recently reported artificial optoelectronic synapses employing QDs as charge-trapping centers (CTCs)

**QDs for CTCs**	**Block layer**	**Excitatory spike** λ (irradiance)	**Inhibitory spike** V_G	**Synaptic functions**	**Ref.**
$CuInSe_2$	PMMA	365 nm (0.06 mW/cm^2) 500 nm, 850 nm	-35 V	1) PPF<br>2) SRDP<br>3) Potentiation-depression cycle<br>4) Rehearsal-induced consolidation<br>5) Dynamic filtering<br>6) Pattern recognition	[1]
PbS	PMMA	365 nm (0.35 mW/cm^2) 550 nm 850 nm	-10 V	1) PPF<br>2) Potentiation-depression cycle<br>3) Rehearsal-induced consolidation<br>4) STM-to-LTM transition<br>5) Associative learning<br>6) Dynamic filtering	[2]
BP	Al_2O_3	473 nm (0.24 mW/cm^2)	4.5 V	1) PPF<br>2) Potentiation-depression cycle<br>3) Associative learning	[3]
$CsPbBr_3$	Al_2O_3	490 nm (0.25 mW/cm^2)	-15 V	1) PPF<br>2) Potentiation-depression cycle<br>3) Rehearsal-induced consolidation<br>4) STM-to-LTM transition	[4]
Carbon dots	Silk protein	365 nm (0.15 mW/cm^2)	-10 V	1) PPF/PPD<br>2) STDP<br>3) Potentiation-depression cycle<br>4) STM-to-LTM transition<br>5) Pattern recognition	[5]
$CsPbBr_3$	PMMA	365 nm (0.15 mW/cm^2)	-10 V	1) PPF<br>2) SRDP	[6]

		450 nm, 520 nm, 660 nm		3) Potentiation-depression cycle<br>4) STM-to-LTM transition	
InP/ZnS	None	405 nm	-40 V	1) Potentiation-depression cycle	[7]
Si	None	1342 nm ($30\ mW/cm^2$)	100 mV	1) PPF<br>2) STDP<br>3) Potentiation-depression cycle<br>4) Pattern recognition<br>5) Aversion learning<br>6) Logic functions	[8]
$CsPbBr_3$	None	445 nm ($100\ mW/cm^2$)	-30 V	1) PPF<br>2) STM-to-LTM transition<br>3) Potentiation-depression cycle<br>4) Pattern recognition	[9]
InP/ZnSe	None	405 nm ($5.14\ mW/cm^2$)	-10 V	1) PPF<br>2) SRDP<br>3) STM-to-LTM transition<br>4) Potentiation-depression cycle<br>5) Rehearsal-induced consolidation<br>6) Associative learning	This work

Reference:

[1] J. Y. Zhang, Z. Y. Guo, T. R. Sun, P. Guo, X. Liu, H. Y. Gao, S. L. Dai, L. Z. Xiong, J. Huang, SmartMat 2023, 14.

[2] J. Zhang, P. Guo, Z. Guo, L. Li, T. Sun, D. Liu, L. Tian, G. Zu, L. Xiong, J. Zhang, J. Huang, Advanced Functional Materials 2023, 33, 2302885.

[3] J.-L. Meng, T.-Y. Wang, L. Chen, Q.-Q. Sun, H. Zhu, L. Ji, S.-J. Ding, W.-Z. Bao, P. Zhou, D. W. Zhang, Nano Energy 2021, 83, 105815.

[4] L. Li, X.-L. Wang, J. Pei, W.-J. Liu, X. Wu, D. W. Zhang, S.-J. Ding, Science China Materials 2020, 64, 1219.

[5] Z. Lv, M. Chen, F. Qian, V. A. L. Roy, W. Ye, D. She, Y. Wang, Z. X. Xu, Y. Zhou, S. T. Han, Advanced Functional Materials 2019, 29.

[6] Y. Wang, Z. Lv, J. Chen, Z. Wang, Y. Zhou, L. Zhou, X. Chen, S. T. Han, Advanced Materials 2018, 30.
[7] H. hu, G. Wen, J. Wen, L. B. Huang, M. Zhao, H. Wu, Z. Sun, Advanced Science 2021, 8, 2100513.
[8] L. Yin, C. Han, Q. Zhang, Z. Ni, S. Zhao, K. Wang, D. Li, M. Xu, H. Wu, X. Pi, D. Yang, Nano Energy 2019, 63, 103859.
[9] G. K. Gupta, I.-J. Kim, Y. Park, M.-K. Kim, J.-S. Lee, ACS Applied Materials & Interfaces 2023, 15, 18055.
